# Supplementary material for: Recombinant Glycoprotein E of Varicella Zoster Virus Contains Glycan-Peptide Motifs That Modulate B Cell Epitopes into Discrete Immunological Signatures
Source: Int J Mol Sci. 2019 Feb 22;20(4):954. doi: 10.3390/ijms20040954 (PMC6412795; doi:10.3390/ijms20040954)
Supplement: Supplementary file 1 [file ijms-20-00954-s001.pdf]

Table S1. Determined N- and O-linked glycosylation sites within glycoprotein E (gE_{HP})

Start	End	Sequence	Glycan modification(s)
73	85	KAYDHN**<u>S</u>**PYIWPR	None; **HexHexNAcNeuAc(2)**
74	85	AYDHN**<u>S</u>**PYIWPR	None; **HexHexNAcNeuAc**
114	145	LMQPTQMSAQEDLGDDTGIHVIPTLNGDDRHK	2 HexHexNAcNeuAc; 2 HexHexNAcNeuAc(2)
114	145	LMQPTQMSAQEDLGDDTGIHVIPTLNGDDRHK	HexHexNAcNeuAc and HexHexNAcNeuAc(2)
114	145	LMQPTQMSAQEDLGDDTGIHVIPTLNGDDRHK	HexHexNAcNeuAc(2)Ac and HexHexNAcNeuAc(2)
114	145	LMQPTQMSAQEDLGDDTGIHVIPTLNGDDRHK	HexHexNAcNeuGc and HexHexNAcNeuAc(2)
153	159	QYGDVFK	None
153	165	QYGDVFKGDLNPK	None
153	170	QYGDVFKGDLNPKPQGQR	None
160	170	GDLNPKPQGQR	None
249	260	MDSPHEYGTWVR	None
305	320	GSDGTSTYATFLVTWK	None
305	324	GSDGTSTYATFLVTWKGDEK	None
321	337	GDEKTRNP**<u>T</u>**PAV**<u>T</u>**PQPR	**2 HexHexNAcNeuAc;** HexHexNAc and HexHexNAcNeuAc
325	337	TRNPTPAV**<u>T</u>**PQPR	**HexHexNAc; HexHexNAcNeuAc; HexHexNAcNeuAc(2)**
325	337	TRNP**<u>T</u>**PAV**<u>T</u>**PQPR	**2 HexHexNAc**; **2 HexHexNAcNeuAc**
325	337	TRNP**<u>T</u>**PAV**<u>T</u>**PQPR	**HexHexNAc**[a] and **HexHexNAcNeuAc**[a]
325	337	TRNP**<u>T</u>**PAV**<u>T</u>**PQPR	**HexHexNAcNeuAc**[a] and **HexHexNAcNeuAc(2)**[a]
430	440	QNCEHAD**N**YTA	dHex(1)Hex(5)HexNAc(4)
430	441	QNCEHAD**N**YTAY	dHex(1)Hex(5)HexNAc(4)
433	441	EHAD**N**YTAY	dHex(1)Hex(5)HexNAc(4)
433	440	EHAD**N**YTA	dHex(1)Hex(5)HexNAc(4)
430	439	QNCEHAD**N**YT	dHex(1)Hex(5)HexNAc(4)NeuAc(1)
430	440	QNCEHAD**N**YTA	dHex(1)Hex(5)HexNAc(4)NeuAc(1)
433	439	EHAD**N**YT	dHex(1)Hex(5)HexNAc(4)NeuAc(1)
433	441	EHAD**N**YTAY	dHex(1)Hex(5)HexNAc(4)NeuAc(1)
433	440	EHAD**N**YTA	dHex(1)Hex(5)HexNAc(4)NeuAc(1)
508	523	GFPPTAGQPPA**<u>T</u>**TKPK	**HexHexNAcNeuAc; HexHexNAcNeuAc(2)**
508	523	GFPP**<u>T</u>**AGQPPA**<u>T</u>**TKPK	**2 HexHexNAcNeuAc**
508	537	GFPPTAGQPPATTKPKEITPVNPGTSPLLR	2 HexHexNAcNeuAc(2)
508	537	GFPP**<u>T</u>**AGQPPA**<u>TT</u>**KPKEITPVNPGTSPLLR	**3 HexHexNAcNeuAc**
508	537	GFPPTAGQPPATTKPKEITPVNPGTSPLLR	HexHexNAcNeuAc; HexHexNAcNeuAc(2)
508	537	GFPPTAGQPPATTKPKEITPVNPGTSPLLR	HexHexNAcNeuAc and HexHexNAcNeuAc(2)
508	537	GFPPTAGQPPATTKPKEITPVNPGTSPLLR	4-6 HexHexNAcNeuAc
524	537	EI**<u>T</u>**PVNPGTSPLLR	None; **HexHexNAcNeuAc**; HexHexNAcNeuAc(2)
524	537	EITPVNPGTSPLLR	HexHexNAcNeuAc and HexHexNAcNeuAc(2)

Bold and underlined Ser/Thr residues were identified as glycosylation sites from the ETD spectra analysis. The corresponding structures in bold were assigned to those sites. Non-bold structures were only identified in the HCD spectra analysis. [a] denotes that both glycans were pinpointed to each site. N-glycopeptides were identified from the pronase sample.

Table S2. Reactivity of VZV-positive and VZV-negative sera towards a panel of synthetic peptides/glycopeptides representing VZV gE[1].

Range of sequence	Complete peptide sequence	Number of VZV+ sera showing reactivity[2]	Number of VZV- sera showing reactivity[3]	Glycan modification verified in gE_{HP}
W64 – Y81	WVNRGESSRKAYDHNSPY	3	1	n.a.
W64 – Y81	WVNRGES*S*RKAYDHNSPY	0	0	-
W64 – Y81	WVNRGES*S*RKAY*DHNSPY	0	1	-
W64 – Y81	WVNRGESS*RKAYDHNSPY	0	0	-
W64 – Y81	WVNRGESSRKAYDHNS*PY	0	1	+
W64 – Y81	WVNRGESSRKAY*DHNSPY	0	2	-
W64 – Y81	WVNRGES*SRKAYDHNSPY	2	2	-
K73 – G90	KAYDHNSPYIWPRNDYDG	10	4	n.a.
K73 – G90	KAY*DHNSPYIWPRNDYDG	2	1	-
K73 – G90	KAYDHNS*PYIWPRNDYDG	1	0	+
K73 – G90	KAYDHNSPYIWPRNDY*DG	1	1	-
K73 – G90	KAYDHNSPY*IWPRNDYDG	1	1	-
V101 – M120	VYNQGRGIDSGERLMQPTQM	4	0	n.a.
V101 – M120	VYNQGRGIDSGERLMQPT*QM	0	0	+[#]
G111 – T130	GERLMQPTQMSAQEDLGDDT	0	0	n.a.
G111 – T130	GERLMQPT*QMSAQEDLGDDT	0	0	+[#]
G111 – T130	GERLMQPT*QMS*AQEDLGDDT	0	1	+[#]
G111 – T130	GERLMQPTQMS*AQEDLGDDT	2	0	+[#]
S121 - G140	SAQEDLGDDTGIHVIPT*LNG	3	0	+
S121 - G140	S*AQEDLGDDTGIHVIPT*LNG	3	2	+[#]
S121 - G140	S*AQEDLGDDTGIHVIPTLNG	3	0	+[#]
S121 - G140	SAQEDLGDDTGIHVIPTLNG	3	0	n.a.

G131 – D150	GIHVIPT*LNGDDRHKIVNVD	0	2	+
G131 – D150	GIHVIPTLNGDDRHKIVNVD	0	0	n.a.
R190 – T207	RIYGVRYTETWSFLPS*LT	0	0	-
T199 – I216	TWSFLPS*LTCTGDAAPAI	1	1	-
L316 – T333	LVTWKGDEKTRNPT*PAVT	0	1	+
T329 – W344	TPAVT*PQPRGAEFHMW	0	0	+
T325 – H342	TRNPTPAVTPQPRGAEFH	0	0	n.a.
T325 – H342	TRNPT*PAVTPQPRGAEFH	0	1	+
T325 – H342	TRNPTPAVT*PQPRGAEFH	0	0	+
L379 – C396‡	LYVPIDPT*CQPMRLYSTC	8	9	-
L406 – R423	LSHMNSGCTFTS*PHLAQR	1	0	-
L406 – R423	LSHMNSGCTFTSPHLAQR	0	0	n.a.
L406 – R423	LSHMNSGCTFT*SPHLAQR	0	0	-
A421 – A440	AQRVASTVYQNCEHADNYTA	1	0	n.a.
N431 – P450	NCEHADNYTAYCLGISHMEP	0	0	n.a.
C442 – G459	CLGISHMEPS*FGLILHDG	0	0	-
G460 – V477	GTTLKFVDTPESLS*GLYV	4	1	-
G460 – V477	GTTLKFVDTPESLSGLYV	0	0	n.a.
G460 – V477	GTTLKFVDTPES*LSGLYV	0	0	-
G460 – V477	GTTLKFVDT*PESLSGLYV	0	0	-
E487 – I504	EAVAYTVVSTVDHFVNAI	0	2	n.a.
E487 – I504	EAVAYTVVS*TVDHFVNAI	0	0	-
E487 – I504	EAVAYTVVST*VDHFVNAI	0	4	-
H499 – G514	HFVNAIEERGFPPT*AG	0	0	+
E505 – P522	EERGFPPTAGQPPATT*KP	1	1	+
E505 – P522	EERGFPPTAGQPPATTKP	0	0	n.a.
E505 – P522	EERGFPPT*AGQPPATTKP	0	2	+
E505 – P522	EERGFPPTAGQPPAT*TKP	4	3	+

R507 – P522	RGFPPTAGQPPAT*TKP	1	0	+
R507 – P522	RGFPPTAGQPPATT*KP	0	0	+
R507 – P522	RGFPPT*AGQPPATTKP	1	1	+
R507 – P522	RGFPPTAGQPPATT*KP	0	1	+
G514 – G531	GQPPAT*TKPKEITPVNPG	3	2	+
G514 – G531	GQPPATTKPKEITPVNPG	4	3	n.a.
G514 – G531	GQPPATT*KPKEITPVNPG	0	2	+
G514 – G531	GQPPATTKPKEIT*PVNPG	4	4	+
Q515 – G531	QPPATT*KPKEITPVNP	0	0	+
Q515 – G531	QPPAT*TKPKEITPVNP	0	0	+
Q515 – G531	QPPATTKPKEIT*PVNP	1	1	+
K523 – Y538	KEIT*PVNPGTSPLLRY	3	1	+
K523 – A540	KEITPVNPGTS*PLLRYAA	3	2	+
K523 – A540	KEITPVNPGT*SPLLRYAA	0	2	+
K523 – A540	KEITPVNPGTSPLLRYAA	3	2	n.a.

[1] The synthetic peptides were immobilized on a glass slide and incubated with sera from VZV positive (individual serum samples n=11 and pooled serum samples n=2) and negative (individual serum samples n=11 and pooled serum samples n=2) individuals and the intensity of fluorescence determined. Serum samples with a relative fluorescence value higher than two standard deviations over the mean of the control group were designated positive. The portion of gE spanning W64–D135 was previously shown to constitute a linear B cell epitope [30]. [2] Serum samples were assigned as VZV-positive using ELISA and immunofluorescence microscopy. [3] Serum samples that did not show reactivity with ELISA nor immunofluorescent microscopy were determined as VZV-negative. n.a. = not assigned, [*] indicates addition of a GalNAc residue to Ser, Thr or Tyr, [#] indicate that the glycosylation sites could not be unambiguously assigned, [‡] the peptide was synthesized with a GalNAc residue present at Thr-386 but this modification was not observed in gE_{HP} or gE_{L} as determined by LC-MS/MS indicating that the observed reactivity could represent cross reactivity with other viral or human proteins

Fig. S1

1	MGTVNKPVVG	VLMGFGIITG	TLRITNPVRA	SVLRYDDFHI	DEDKLDTNSV
51	YEPYYHSDHA	ESSWVNRGES	SRKAYDHNSP	YIWPRNDYDG	FLENAHEHHG
101	VYNQGRGIDS	GERLMQPT*QM	S*AQEDLGDDT	GIHVIPTLNG	DDRHKIVNVD
151	QRQYGDVFKG	DLNPKPQGQR	LIEVSVEENH	PFTLRAPIQR	IYGVRYTETW
201	SFLPSLTCTG	DAAPAIQHIC	LKHTTCFQDV	VVDVDCAENT	KEDQLAEISY
251	RFQGKKEADQ	PWIVVN**TSTL	FDELELDPPE	IEPGVLKVLR	TEKQYLGVYI
301	WNMRGSDGTS	TYATFLVTWK	GDEKTRNPTP	AVTPQPRGAE	FHMWNYHSHV
351	FSVGDTFSLA	MHLQYKIHEA	PFDLLLEWLY	VPIDPTCQPM	RLYSTCLYHP
401	NAPQCLSHMN	SGCTFTSPHL	AQRVASTVYQ	NCEHADNYTA	YCLGISHMEP
451	SFGLILHDGG	TTLKFVDTPE	SLSGLYVFVV	YFNGHVEAVA	YTVVSTVDHF
501	VNAIEERGFP	PTAGQPPATT	KPKEITPVNP	GTSPLLRYA	

TRYPSIN, **PRONASE**, **BOTH.** *One of these is glycosylated. ** Not observed but possible N-glycosylation site.

Fig. S2

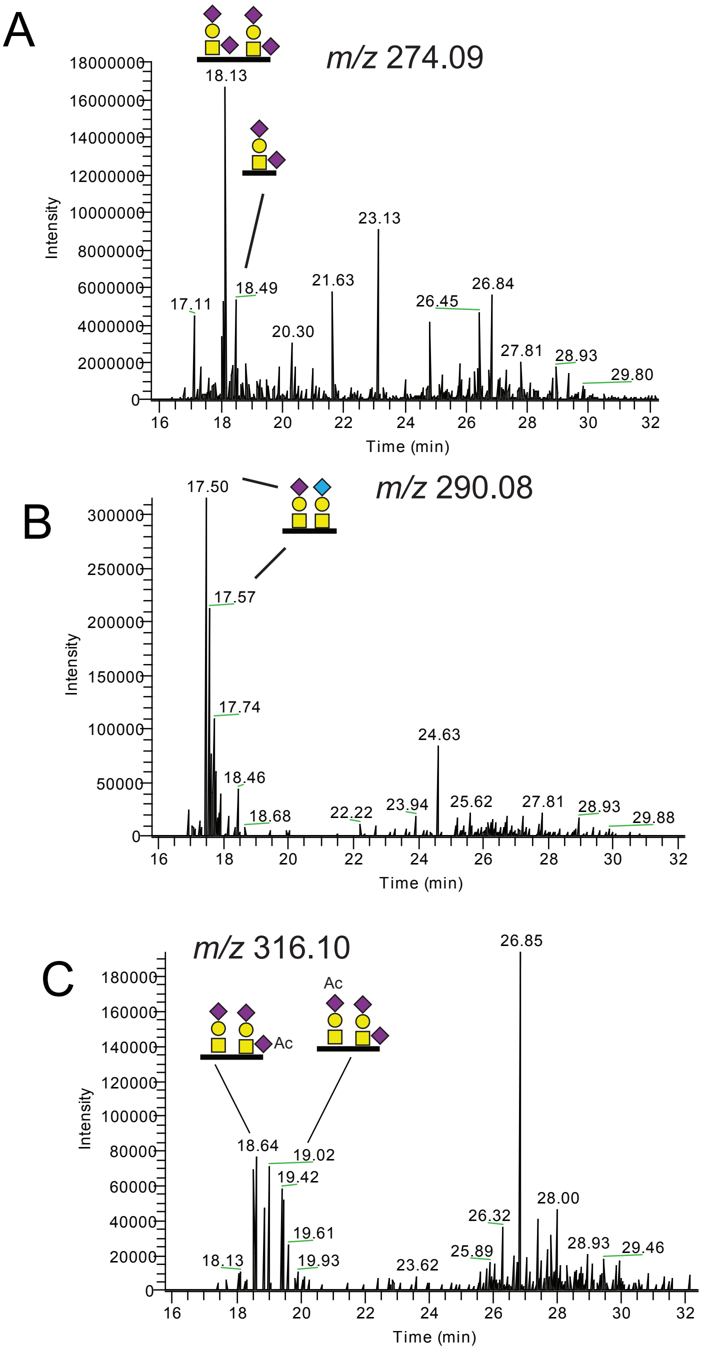
A
m/z 274.09
Intensity
Time (min)
18.13
18.49
17.11
20.30
21.63
23.13
26.45
26.84
27.81
28.93
29.80
B
m/z 290.08
Intensity
Time (min)
17.50
17.57
17.74
18.46
18.68
22.22
23.94
24.63
25.62
27.81
28.93
29.88
C
m/z 316.10
Intensity
Time (min)
26.85
Ac
18.64
19.02
19.42
19.61
19.93
18.13
23.62
25.89
26.32
28.00
28.93
29.46

Fig. S3

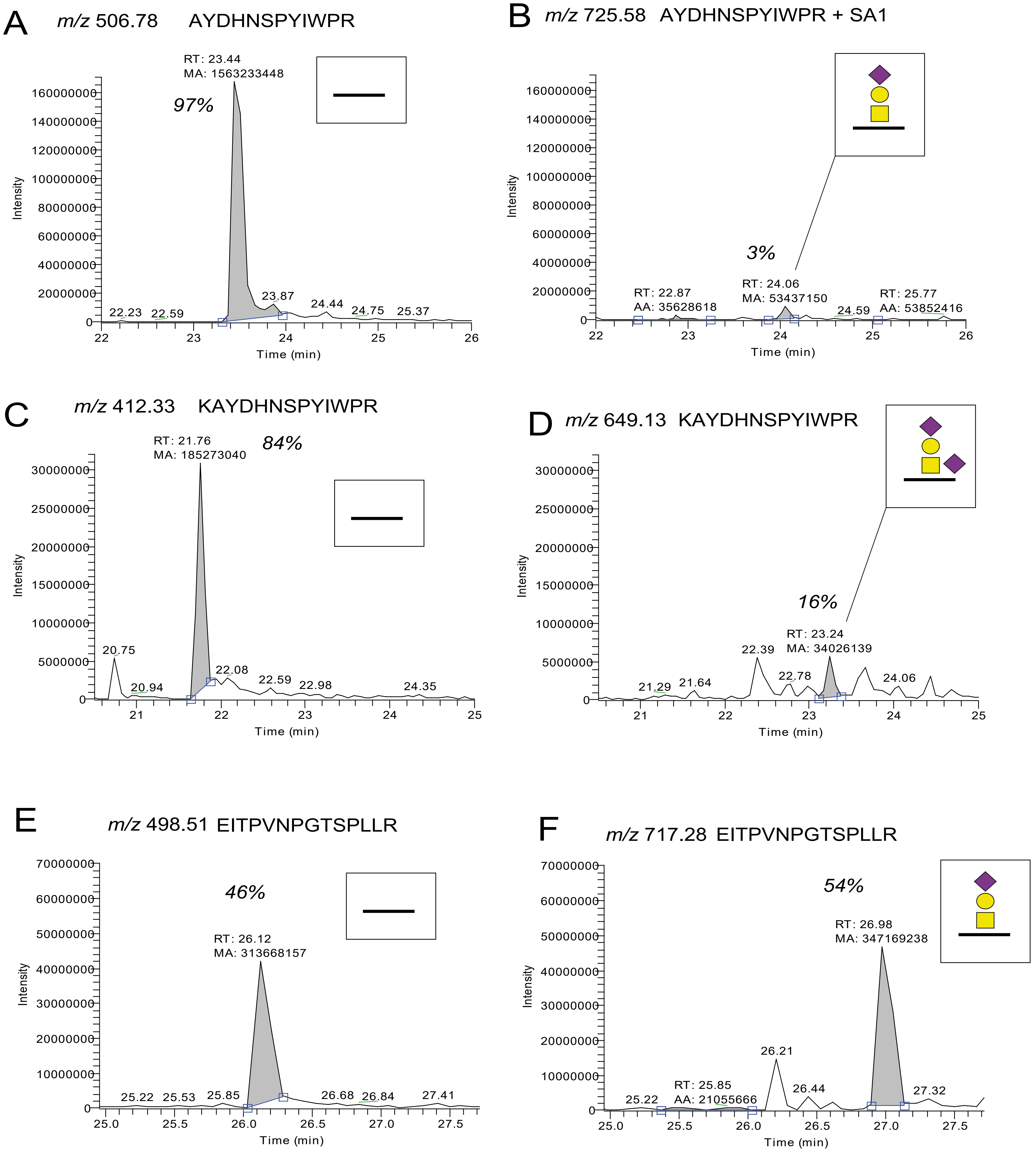

A m/z 506.78 AYDHNSPYIWPR
RT: 23.44
MA: 1563233448
97%
B m/z 725.58 AYDHNSPYIWPR + SA1
3%
RT: 24.06
MA: 53437150
RT: 22.87
AA: 35628618
RT: 25.77
AA: 53852416
C m/z 412.33 KAYDHNSPYIWPR
RT: 21.76
MA: 185273040
84%
D m/z 649.13 KAYDHNSPYIWPR
16%
RT: 23.24
MA: 34026139
E m/z 498.51 EITPVNPGTSPLLR
46%
RT: 26.12
MA: 313668157
F m/z 717.28 EITPVNPGTSPLLR
54%
RT: 26.98
MA: 347169238
RT: 25.85
AA: 21055666
Intensity
Time (min)

Fig. S4

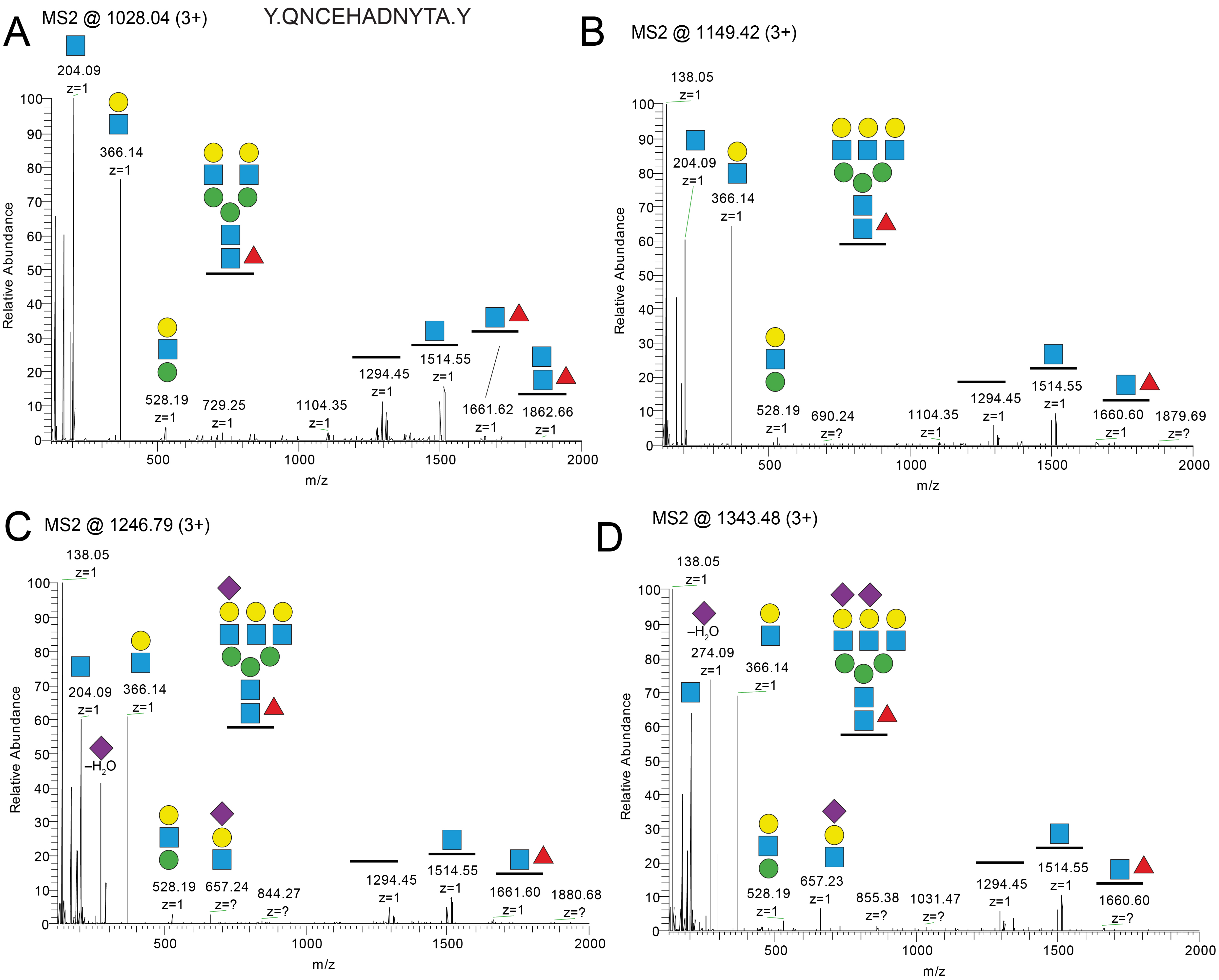

A
MS2 @ 1028.04 (3+)
Y.QNCEHADNYTA.Y
204.09
z=1
366.14
z=1
528.19
z=1
729.25
z=1
1104.35
z=1
1294.45
z=1
1514.55
z=1
1661.62
z=1
1862.66
z=1
Relative Abundance
m/z
B
MS2 @ 1149.42 (3+)
138.05
z=1
204.09
z=1
366.14
z=1
528.19
z=1
690.24
z=?
1104.35
z=1
1294.45
z=1
1514.55
z=1
1660.60
z=1
1879.69
z=?
Relative Abundance
m/z
C
MS2 @ 1246.79 (3+)
138.05
z=1
204.09
z=1
366.14
z=1
−H2O
528.19
z=1
657.24
z=?
844.27
z=?
1294.45
z=1
1514.55
z=1
1661.60
z=1
1880.68
z=?
Relative Abundance
m/z
D
MS2 @ 1343.48 (3+)
138.05
z=1
−H2O
274.09
z=1
366.14
z=1
528.19
z=1
657.23
z=1
855.38
z=?
1031.47
z=?
1294.45
z=1
1514.55
z=1
1660.60
z=?
Relative Abundance
m/z

Fig. S5

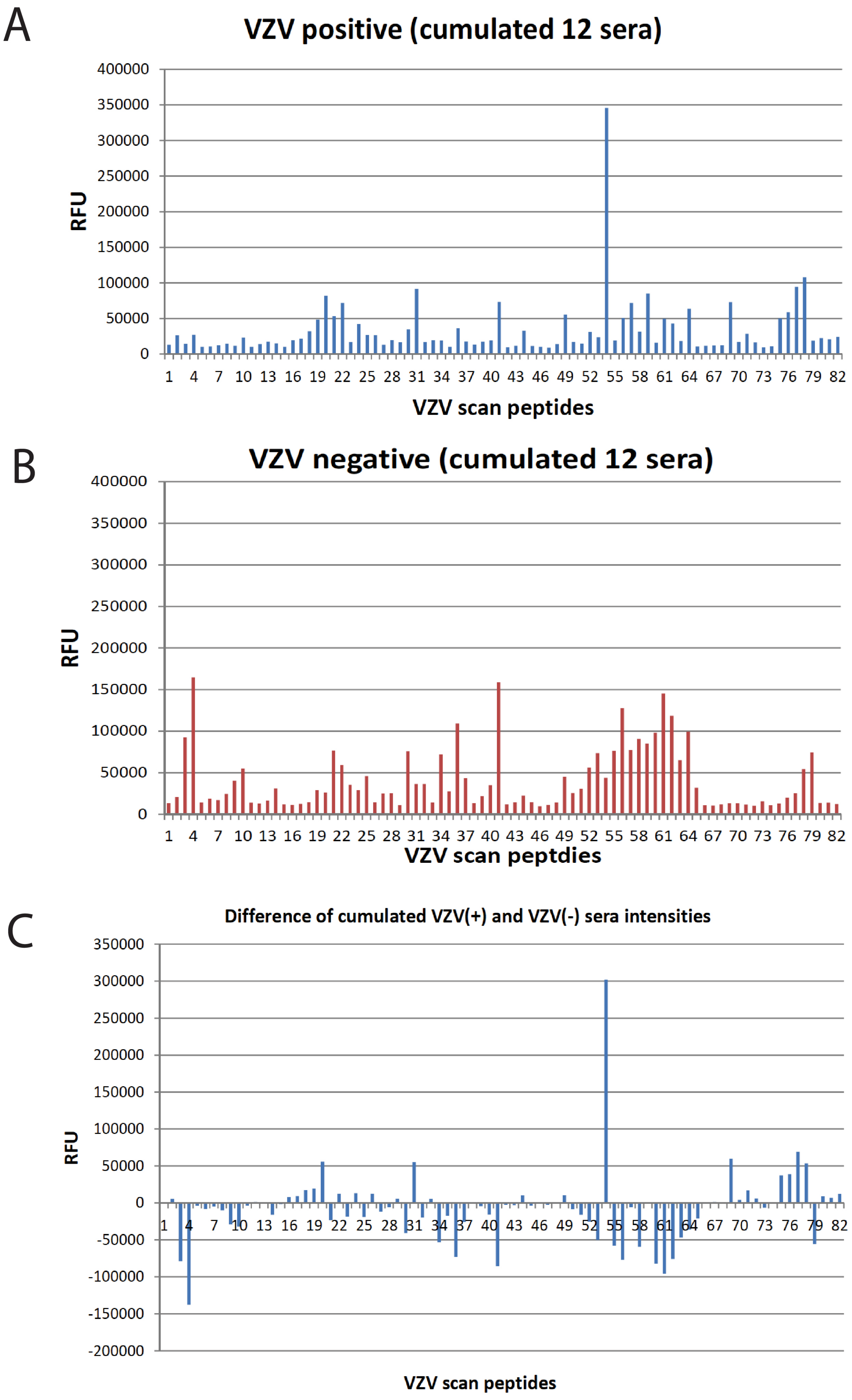

A
VZV positive (cumulated 12 sera)
RFU
400000
350000
300000
250000
200000
150000
100000
50000
0
1 4 7 10 13 16 19 22 25 28 31 34 37 40 43 46 49 52 55 58 61 64 67 70 73 76 79 82
VZV scan peptides
B
VZV negative (cumulated 12 sera)
RFU
400000
350000
300000
250000
200000
150000
100000
50000
0
1 4 7 10 13 16 19 22 25 28 31 34 37 40 43 46 49 52 55 58 61 64 67 70 73 76 79 82
VZV scan peptdies
C
Difference of cumulated VZV(+) and VZV(-) sera intensities
RFU
350000
300000
250000
200000
150000
100000
50000
0
-50000
-100000
-150000
-200000
1 4 7 10 13 16 19 22 25 28 31 34 37 40 43 46 49 52 55 58 61 64 67 70 73 76 79 82
VZV scan peptides
